# Evolution of vaccination rates after the implementation of a free systematic pneumococcal vaccination in Catalonian older adults: 4-years follow-up

**DOI:** 10.1186/1471-2458-6-231

**Published:** 2006-09-18

**Authors:** Angel Vila-Córcoles, Olga Ochoa-Gondar, Francisco Ester, Nuria Sarrá, Xabier Ansa, Neus Saún, EVAN Study Group

**Affiliations:** 1 Primary Care Service of Tarragona-Valls. EVAN Study Group. Catalonian Health Institute. District of Tarragona. Catalonia, Spain

## Abstract

**Background:**

The systematic vaccination with 23-valent polysaccharide pneumococcal vaccine (PPV) was introduced as a strategic objective of health for all the people over 65 in Catalonia in 1999. We analysed the evolution of the pneumococcal vaccination rates from 2000 to 2003.

**Methods:**

We conducted a retrospective population-based study including all the individuals 65 years or older assigned to 8 Primary Care Centres (PCCs) in Tarragona (Catalonia, Spain), who figured in the administrative population databases on 31 December 2003 (n = 10,410 persons). We assessed whether every person had received PPV during the last four years (2000 to 2003) or whether they had received it before January 2000. Data sources were the computerised clinical records of the 8 participating PCCs, which included adult vaccination registries and diagnoses coded of International Classification of Diseases 9^th ^Review

**Results:**

The overall vaccination uptake increased to 38.6% at the end of 2000. Global accumulated coverages increased more slowly the following years: 44.4% in 2001, 50.9% in 2002, and 53.1% at the end of 2003. Vaccine uptake varied significantly according to age (46.7% in people 65–74 years-old, 60.9% in people 75 years or more; p < 0.001) and number of diseases or risk factors (DRFs) for pneumonia (47.1% vaccinated in people without DRFs, 56.8% in patients with one DRF, and 62.2% in patients with two or more DRFs; p < 0.001). The highest coverages were observed among those patients with: diabetes (65.9%), active neoplasia (64.8%), history of stroke (63.7%), and chronic lung disease (63.5%). The lowest uptake was observed among smokers (48.7%).

**Discussion:**

The pneumococcal vaccination coverage increased quickly after the introduction of the recommendation for free vaccination in all the elderly people (with and without risk factors), but two years after the improvement the coverage became stable and increased slowly.

## Background

Infections caused by *Streptococcus pneumoniae *are an important cause of morbidity and mortality in the elderly, especially in those with chronic diseases [1]. The 23-valent polysaccharide pneumococcal vaccine (PPV) has been available since 1983 and is currently recommended for use in the elderly and high risk groups. More than 20 years later, despite many clinical trials and observational studies on this vaccine, its effectiveness is controversial. Several observational trials have shown a protective effect in preventing pneumococcal bacteraemia, but others have not shown this effect.

Meta analyses carried out regarding this vaccine are not conclusive [2-5]. The last published Cochrane review concluded that the PPV may not be effective to prevent pneumonia, but the vaccine has shown an efficacy of at least 53% to prevent pneumococcal invasive disease [6]. Nowadays many advisory committees on immunisation practices recommend the use of PPV in high risk groups and individuals over the age of 65 years on the basis that the effectiveness of PPV in preventing pneumococcal bacteraemia among the elderly and high risk adults has been demonstrated in observational studies. The vaccination is cost-effective and there is sufficient evidence to justify its widespread use [7-9].

In Spain, pneumococcal vaccination is not uniformly recommended in all the Spanish Autonomous Regions. In Catalonia, a region in the northeast of Spain with a population of 6 million people, a nationwide pneumococcal vaccination program for all people 65 years or older (with or without risk factors) began in October 1999. Before this date PPV only was prescribed in hospital or outpatient visits for high-risk subjects, and pneumococcal vaccination coverage was 5.8% among our general elderly population [10].

In the present study, we assessed the evolution of pneumococcal vaccination coverage from 2000 to 2003 in the general elderly population.

## Methods

We conducted a retrospective, multicentred population-based study located in The Primary Care Service of Tarragona (Catalonia, Spain).

In the Spanish Health Care System, all persons are assigned to one Primary Care Centre (PCC) and a General Practitioner. All relevant medical details are filed on patients from primary care visits, immunisations, medical prescriptions, laboratory tests, hospital admissions, and visits to outpatient clinics. In Catalonia all individuals are also assigned to a PCC. In the Health District of Tarragona (an urban area), the Catalonian Health Institute have 12 PCCs with an overall assigned population of 134,232 inhabitants (19 833 persons 65 years or older).

We included in the present study all the persons aged 65 years or older, who are assigned to 8 of the 12 PCCs and who had at least 1 year of recorded database history prior to the start of the survey to determine medical conditions and vaccination status. The eligible population thus included 10,410 persons 65 yrs or older on December 31, 2003, of which 4481 were male and 5929 female, with a median age of 74.6 yrs (SD: 7.5). The mean age of eligible subjects in 8 participating PCC ranged between 73.6 and 75.7 years-old.

The selection of the 8 participating PCCs was not randomised and they were chosen taking into account the existence of electronic clinical registries working since 1998 or before. The other 4 PCCs of the Health District of Tarragona were not included because they only introduced computerised clinical records more recently.

We assessed whether every eligible person had received the pneumococcal vaccine during the last four years (from Jan 1, 2000 to Dec 31, 2003) or whether they had received it before January 2000. The pneumococcal vaccination program was executed by PCCs and the vaccine used was a 23-valent polysaccharide pneumococcal vaccine (PPV). General Practitioners and Primary Care Nurses registered the vaccination date in the electronic medical record.

Data sources were the computerised clinical records of the 8 participating PCCs, which included adult vaccination registries and diagnosis code of international Classification Diseases 9^th ^Review (ICD-9). We considered as having been correctly vaccinated those people who had the PPV specific code registered, together with the date of administration in their clinical record. We also considered in each individual the presence or absence of any code (ICD-9) of the following diseases or risk factors (DRF) for pneumonia: diabetes mellitus, chronic heart disease, chronic lung disease, smoking, history of stroke, chronic nephropathy, chronic liver disease, excessive consumption of alcohol, active neoplasia and immunocompromised status.

Vaccine coverages were calculated according to age-groups, sex and presence of DRFs. The statistical differences between vaccine coverages were evaluated using the differences between the percentages. Odds ratio (OR) were used to evaluate the association between the reception of pneumococcal vaccine and the presence of each DRF. All results were expressed with 95% confidence intervals (CIs).

The study was approved by the ethical committee of the Catalonian Health Institute (expedient FIS PI021117) and was conducted in accordance with the general the general principles for observational studies.

## Results

The overall pneumococcal vaccine coverage increased to 38.6% at the end of the first year after the implementation of the systematic recommendation to receive the PPV in all elderly individuals. The overall accumulative coverages increased more slowly in the following years: 44.4% in 2001, 50.9% in 2002, and 53.1% in 2003. At the end of 2003, pneumococcal vaccine coverages ranged from 44.9% to 66.3% in 8 participating PCCs.

The greater number of pneumococcal vaccines were administered during influenza vaccination campaigns. 63.2% of PPV was administered in October and November, 23.6% of PPV was administered during the cold months (December-April) and 13.2% during the warm months (May-September).

At the end of 2003, pneumococcal vaccine coverages varied significantly according to age: 46.7% (95% CI: 45.5–48.0) among people 65–74 years-old, and 60.9% (95% CI: 58.9–61.8) among people 75 years or older (p < 0.001). The vaccination coverage was 53.4% (95% CI: 51.9–54.9) in males and 52.7% (95% CI: 51.4–54.0) in females (p = 0.481). The vaccination coverages in December 2003 according to age and gender are shown in Table 1.

The evolution of vaccination coverages during the four years of the survey, according to the presence of diseases or risk factors (DRF) for pneumonia are shown in Table 2. At the end of the survey, we observed higher PPV coverages among those patients with diabetes (65.9%), active malignancy (64.8%), history of stroke (63.7%), and chronic lung disease (63.5%). The lowest PPV rates were observed among smokers (48.7%), drinkers (54.5%) and patients with chronic liver disease (53.1%).

We observed a significant increase of pneumococcal vaccination coverage according to the number of DRFs for pneumonia: 47.1% vaccinated among those without DRF, 56.8% among those with one DRF, and 62.2% among those with two or more DRFs for pneumonia (p < 0.001).

Table 3 shows pneumococcal vaccination coverage in the study population at the end of 2003 according to the presence or absence of each disease or risk factor for pneumonia. Except in three conditions (chronic liver disease, consumption of alcohol and smoking) the pneumococcal vaccine coverages were significantly higher among those patients who had a DRF in comparison to those who did not have a DRF.

## Discussion

The 23-valent polysaccharide pneumococcal vaccine (PPV) for the elderly population has been recommended in some developed countries for more than a decade. Nevertheless, despite the time that has elapsed, achieving a high population uptake has proven to be difficult [11-14].

In Catalonia the recommendation for systematic pneumococcal vaccination in the general elderly population was introduced at the end of 1999 as an objective for all the Primary Care Centers (PCC) of the Catalonian Health Service. Before this date, the prescription of pneumococcal vaccination was only possible in hospital or during a visit to a specialist clinics for high risk subjects (diabetes, chronic heart or lung disease, and inmunocompromised subjects). Before starting the vaccination program, pneumococcal vaccination coverage reached about 6% among our general elderly population [10]. We analysed the evolution of pneumococcal vaccine uptake since December 2000 to December 2003. During this period of time no specific campaign was carried out, and the invitation for pneumococcal vaccination was offered when the elderly subjects (with or without risk factors) came to the PCCs during the influenza vaccination campaigns or in any other visit throughout the rest of the year.

In our study population, the vaccination coverage increased quickly after the introduction of the recommendation for free vaccination in all the elderly people (from 6% at the beginning of 2000 to 44% at the end of 2001). But two years later the improvement of coverage became slow (51% in 2002 and 53% in 2003).

Although the sample was wide (more than 10,400 elderly individuals), all the persons belong to one of 8 PCCs located in the district of Tarragona. This could be a problem if the results were to be extrapolated exactly to the whole of Catalonia. The 8 PCCs included in our study were not randomised. They were chosen because their computerised data registers had been working for more than 6 years and they represented the 12 PCCs of the district of Tarragona.

Although 8 participating PCCs had electronic registries working before starting the program, it is possible that some vaccinations given prior to the existence of the computerized registries would not have been recorded in the current computerized system.

We think that our current coverage (53%) can be considered low, this was achieved four years after the introduction of the recommendation for systematic pneumococcal vaccination in all elderly people. The low coverage among 65–74 year olds could be due in part to a limited opportunity for persons turning 65 to have received the pneumococcal vaccine. Age was calculated by age at the end of the year and consequently persons who turned 65 near the end of the year had little opportunity to receive the PPV.

In order to evaluate our results, it must be known that in Spain, the medical care is a publically funded service for all the population and most prescriptions (including PPV) are free for people over 65 years (there is a 60% discount for people under 65 yrs) and this gives an important difference when comparing vaccination uptakes with other countries where medical care and prescriptions need to be paid, totally or partially, directly or through a private medical insurance.

The vaccination coverages in developed countries where PPV use has been recommended for a longer period of time are about 50% to 65% in the overall elderly population [11-14]. We think that the doubts about the effectiveness of PPV could have influenced clinical practice and this could have been the reason why a higher coverage with PPV was not reached. Primary Care physicians (they are the principal promoters of the vaccination) should be made aware that although meta analyses carried out about this vaccine were not conclusive, this does not mean that the vaccine is not effective, since several observational studies have proven its efficacy and cost-effectiveness in preventing invasive pneumococcal disease.

A meta-analysis limited to studies in more developed countries showed a protective effect to prevent pneumococcal bacteraemia but did not find a significant protective effect against pneumonia among elderly subjects or high-risk groups [5]. A recent Cochrane review [6] concluded that, while polysaccharide pneumococcal vaccines do not appear to reduce the incidence of pneumonia or death in elderly people (with or without chronic diseases), the evidence from non-randomised studies suggests that the vaccines are effective in reducing the incidence of the more specific outcome (invasive pneumococcal disease) among adults and immunocompetent elderly subjects 55 years or older. However, in the last published meta-analysis, Melegaro[15] concluded that the PPV provides 65% protection against invasive pneumococcal disease in the general elderly population, having a moderate 20% effect in the high risk elderly. They also concluded that the vaccine could have a little or null effect against pneumonia in the general elderly population (-20% to 16%). In a recent review published in 2004, Fedson and Liss concluded that the PPV is effective in preventing pneumococcal bacteraemia and invasive pneumococcal disease, the vaccination is cost-effective, and they recommend the vaccination in high-risk individuals and the overall elderly population [16].

In USA the current uptakes are about 60% in the general elderly population [11,12]. In Stockholm County (Sweden), an interventionist vaccination program (with a low cost PPV, support of the media and mailing individually to all the population over 65) achieved only a 37% of coverage in 2 years (1998–99) [13]. In Victoria (Australia) the PPV coverage among people 65 years or older increased from 7% in 1997 to 51% in 2000 after a publically funded pneumococcal vaccination program among all the elderly population [14].

Our study shows that once the mid range vaccination rates are reached, they are hard to improve, even with a free vaccine.

During the four years of the survey, no specific PPV campaign was carried out and the strategy used for the vaccination was done in an opportunistic way. We believe that this strategy probably was not very efficient, since the vaccination rates reached in elderly people with high risk were only slightly more than in those with low risk. Although the last published meta-analysis[15] concluded that the vaccine must be less effective among the population at high-risk of suffering from pneumonia, this is a controversial aspect. We think that these high-risk individuals could have received more benefit from the vaccination because the incidence of pneumococcal infection is higher in comparison with low-risk groups [17].

The vaccine coverages in several countries are compared with difficulty due to the different characteristics of each National Health Service. Our study contributes valuable information to know and to compare the evolution of pneumococcal vaccine coverages in a European Region where a free PPV has been offered to all elderly people (with or without risk factors) since the end of 1999.

Although there weren't any guidelines recommending the simultaneous administration of pneumococcal and influenza vaccines in our country, we observed that there was a correlation between both; since almost 63% of PPV were administrated in our study population during the influenza vaccination campaigns. A pneumococcal vaccine coverage of 90% has been recently set as an objective in all the subjects aged 65 or older by the Center for Disease Control for 2010 [12]. We think that Influenza vaccination campaigns can be a good opportunity to attract those who have not had the PPV. Some studies have shown that there is an additive effect between both vaccines [13,18]. This strategy could be good to increase significantly the PPV coverage.

We believe that some work has to be done to reduce the existing doubts about the effectiveness of the PPV among the health professionals. It is true that there is no documented evidence of an effect of this vaccine on mortality rates in many developed countries; but it would be desirable that similar levels of evidence were required for many other recommended preventive measures, and in particular for therapies that are commonly used.

## Conclusion

The pneumococcal vaccination coverage increased quickly after the introduction of the recommendation for free vaccination in all the elderly people (with and without risk factors), but two years after the improvement the coverage became stable and increased slowly. Our study shows that once the acceptable vaccination rates are reached, they are hard to improve, even with a free vaccine.

## Competing interests

Study supported by Grants from the Health Research Fund (FIS) of the Spanish Ministry of Health and Consumer Affairs (expedients PI021117 and PI050231).

## Authors' contributions

AV, OO and XA designed the study. AV, OO, XA, NSaun, FE, NSarra and ESG assessed outcomes, and wrote and edited the paper. AV coordinated the study. OO, FE, NS and ESG obtained the data.

## Pre-publication history

The pre-publication history for this paper can be accessed here:



## Figures and Tables

**Table 1:** Pneumococcal vaccination coverages at the end of 2003 in people 65 years or older in Tarragona (Catalonia, Spain) according to age groups and gender.

**Gender**	**Male**	**Female**	**Difference (male-female)**			**Overall population**	
**Age groups**	**%**	**%**	**%**	**(95% CI)**	**P-value**	**%**	**(95% CI)**
**65–69 yrs (n = 3189)**	37.5	41.1	-3.6	(-7.0 to -0.2)	0.040	39.3	(37.6 to 41.0)
**70–74 yrs (n = 2814)**	55.8	54.4	1.4	(-2.5 to 5.3)	0.453	55.1	(53.3 to 56.9)
**75–79 yrs (n = 2183)**	65.9	57.3	8.6	(4.5 to 12.7)	0.001	61.6	(59.6 to 63.6)
**80–84 yrs (n = 1335)**	66.7	60.6	6.1	(0.7 to 11.5)	0.027	63.6	(60.8 to 66.4)
**85–89 yrs (n = 609)**	65.8	62.8	3.0	(-6.3 to 12.3)	0.469	64.3	(60.5 to 68.1)
**90 or more yrs (n = 280)**	54.5	54.6	-0.1	(-12.2 to 12.0)	0.980	54.5	(48.7 to 60.3)
**Overall (N = 10,410)**	53.4	52.7	0.7	(-1.2 to 2.6)	0.481	53.1	(52.2 to 54.9)

**Table 2:** Evolution of pneumococcal vaccination coverages from 2000 to 2003 in people 65 years or older in Tarragona (Catalonia, Spain) according to the presence of certain diseases or risk factors (DRF) for pneumonia.

**VARIABLES**	**2000**	**2001**	**2002**	**2003**	
	**%**	**%**	**%**	**%**	**(95% CI)**
**Chronic lung disease**	48.1	54.2	61.5	63.5	(60.8–66.2)
**Diabetes mellitus**	45.1	51.2	58.4	65.9	(64.0–67.8)
**Active neoplasia**	43.1	56.7	62.1	64.8	(59.3–70.3)
**Immunocompromise**	47.1	55.0	61.3	63.1	(60.6–65.6)
**Chronic heart disease**	47.7	53.5	60.4	63.0	(60.4–65.6)
**Chronic nephropathy**	48.5	55.3	60.2	62.7	(58.0–67.4)
**Stroke**	45.5	52.9	62.1	63.7	(58.5–68.9)
**Chronic liver disease**	36.7	46.5	52.1	53.1	(46.6–59.6)
**Drinkers**	36.3	43.6	50.8	54.5	(48.8–60.2)
**Smokers**	32.2	38.9	46.6	48.7	(45.6–51.8)
**Level of risk for pneumonia**					
**None DRF**	33.9	38.9	45.1	47.1	(45.8–48.4)
**One DRF**	41.9	47.9	54.6	56.8	(55.1–58.5)
**Two or more DRF**	45.5	52.6	60.3	62.2	(60.1–64.3)
**Overall population**	38.6	44.4	50.9	53.1	(52.2–54.9)

**Table 3:** Pneumococcal vaccination coverages at December 31, 2003 according to the presence or absence of certain diseases or risk factors (DRF) for pneumonia among people 65 years or older in Tarragona (Catalonia, Spain).

**VARIABLES**	**Percent vaccinated among those with DRF**		**Percent vaccinated among those without DRF**		Odds Ratio*		
	**%**	**(95% CI)**	**%**	**(95% CI)**	**OR**	**(95%CI)**	**P-value**
**Chronic lung disease**	63.5	(60.8–66.2)	51.8	(50.8–52.8)	1.57	(1.38–1.79)	<0.001
**Diabetes mellitus**	65.9	(64.0–67.8)	49.6	(48.5–50.7)	1.52	(1.38–1.67)	<0.001
**Active neoplasia**	64.8	(59.3–70.3)	52.8	(51.8–53.8)	1.62	(1.24–2.10)	<0.001
**Immunocompromise**	63.1	(60.6–65.6)	51.7	(50.7–52.7)	1.56	(1.38–1.76)	<0.001
**Chronic heart disease**	63.0	(60.4–65.6)	51.8	(50.8–52.8)	1.48	(1.30–1.68)	<0.001
**Chronic nephropathy**	62.7	(58.0–67.4)	52.7	(51.8–53.7)	1.37	(1.11–1.71)	0.004
**Stroke**	63.7	(58.5–68.9)	52.8	(51.8–53.7)	1.41	(1.11–1.80)	0.005
**Chronic liver disease**	53.1	(46.6–59.6)	53.1	(52.1–54.1)	0.96	(0.72–1.29)	0.812
**High alcohol consumption**	54.5	(48.8–60.2)	53.1	(52.1–54.1)	1.13	(0.88–1.46)	0.319
**Smoking**	48.7	(45.6–51.8)	53.5	(52.5–54.5)	0.87	(0.75–1.01)	0.073

*Odds Ratio (OR) were calculated for those people who received the pneumococcal vaccination.

## References

[B1] Loeb M (2004). Pneumonia in the elderly. Curr Opin Infect Dis.

[B2] Fine MJ, Smith MA, Carson CA, Meffe F, Sankey SS, Weissfeld LA, Detsky AS, Kapoor WN (1994). Efficacy of pneumococcal vaccination in adults. A meta-analysis of randomized controlled trials. Arch Intern Med.

[B3] Moore RA, Wiffen PJ, Lipsky BA (2000). Are the neumococcal polysaccharide vaccines effective? Meta-analysis of the prospective trials. BCM Family Practice.

[B4] Cornu C, Yzebe D, Leophonte P, Gaillat J, Boissel JP, Cucherat M (2001). Efficacy of pneumococcal polysaccharide vaccine in immunocompetent adults: a meta-analysis of randomized trials. Vaccine.

[B5] Mangtani P, Cutts F, Hall AJ (2003). Efficacy of polysaccharide pneumococcal vaccine in adults in more developed countries: the state of the evidence. Lancet Infect Dis.

[B6] Dear K, Holden J, Andrews R, Tatham D (2003). Vaccines for preventing pneumococcal infection in adults. Cochrane Database Syst Rev.

[B7] Centers for Disease Control and Prevention (1997). Prevention of pneumococcal disease: Recommendations of the advisory committee on immunisation practices (ACIP). MMWR.

[B8] Task Force on Community Preventive Service (2000). Recommendations regarding interventions to improve vaccinations coverage in children, adolescents and adults. Am J Prev Med.

[B9] Salleras Ll, Urbitzondo Ll, Fernández N, Comin E, Sanchez F, Batalla J (2001). Pneumococcal vaccine in the elderly population'[in Spanish]. Med Clin (Barc).

[B10] Vila A, Ochoa O, Hospital I, Bria X, Llor C, Montañés D, Grupo de Estudio EVAN-65 (2004). EVAN-65 project: evaluation of the effectiveness of pneumococcus vaccination in the elderly population over 65.[in Spanish]. Aten Primaria.

[B11] Daniels NA, Nguyen TT, Gildengorin G, Perez-Stable EJ (2004). Adult immunization in university-based primary care and specialty practices. J Am Geriatr Soc.

[B12] Center for Disease Control and Prevention (2004). Influenza and pneumococcal vaccination coverage among persons aged > or = 65 years and persons aged 18–64 years with diabetes or asthma – United States, 2003. MMWR Morb Mortal Wkly Rep.

[B13] Christenson B, Hedlund J, Lundbergh P, Ortqvist A (2004). Additive preventive effect of influenza and pneumococcal vaccines in elderly persons. Eur Respir J.

[B14] Andrews R, Counahan M, Hogg G, McIntyre PB (2004). Effectiveness of a publicy funded pneumococcal vaccination program against invasive pneumococcal disease among the elederly in Victoria, Australia. Vaccine.

[B15] Melegaro A, Edmunds WJ (2004). The 23-valent pneumococcal polysaccharide vaccine. Part I. Efficacy of PPV in the elderly: a comparison of meta-analyses. Eur J Epidemiol.

[B16] Fedson DS, Liss C (2004). Precise answers to the wrong question: prospective clinical trials and the meta-analyses of pneumococcal vaccine in elderly and high-risk adults. Vaccine.

[B17] Dominguez A, Salleras L, Cardenosa N, Ciruela P, Carmona G, Martinez A, Torner N, Fuentes M (2002). The epidemiology of invasive Streptococcus pneumoniae disease in Catalonia (Spain). A hospital-based study. Vaccine.

[B18] Nichol KL (1999). The additive benefits of influenza and pneumococcal vaccinations during influenza seasons among elderly persons with chronic lung disease. Vaccine.

